# Fatal equine gas gangrene associated with *Clostridium septicum* in the Brazilian Amazon: clinicopathological and molecular characterization of a 20-case series

**DOI:** 10.1007/s11259-026-11497-6

**Published:** 2026-09-04

**Authors:** Loise Araújo de Sousa, Laura Rosa Corrêa, Hanna Gabriela da Silva Oliveira, Felipe Masiero Salvarani

**Affiliations:** Laboratório de Medicina Veterinária Preventiva E Vacinologia, Instituto de Medicina Veterinária, Universidade Federal Do Pará, Castanhal, PA 68740-970 Brazil

**Keywords:** Equine gas gangrene, Clostridial myonecrosis, Multiplex PCR, Amazon biome, *Clostridium septicum*

## Abstract

Clostridial gas gangrene is an acute necrotizing infection of skeletal muscle characterized by rapid tissue destruction and severe systemic toxemia in horses; however, systematically documented case series from tropical settings remain scarce. Here, we describe a clinicopathological and molecularly confirmed series of 20 fatal equine gas gangrene cases diagnosed in the eastern Brazilian Amazon (Pará State) between 2018 and 2026. Horses presented with abrupt onset of severe lameness, rapidly progressive swelling of a limb or muscle group, and palpable subcutaneous crepitus, frequently following penetrating trauma, intramuscular injections, or other breaches of tissue integrity. Despite antimicrobial and anti-inflammatory treatment, all animals died or were euthanized due to rapid deterioration and poor prognosis. Necropsy consistently revealed extensive hemorrhagic myonecrosis with serosanguineous exudation and intramuscular/subcutaneous gas accumulation. Histology showed widespread coagulative to floccular myonecrosis, marked interstitial edema and hemorrhage, frequent vascular thrombosis, and abundant intralesional bacillary structures morphologically compatible with clostridia. A multiplex PCR assay targeting major histotoxic clostridia detected *Clostridium septicum* DNA in affected skeletal muscle from all cases, with no amplification of other targets. This case series provides clinically relevant documentation of fatal *C. septicum*–associated myonecrosis in horses raised under tropical conditions and reinforces the importance of early recognition, strict aseptic practices during intramuscular procedures, and integration of pathology with molecular diagnostics for definitive etiological confirmation.

**Graphical Abstract:**

**Figure:**
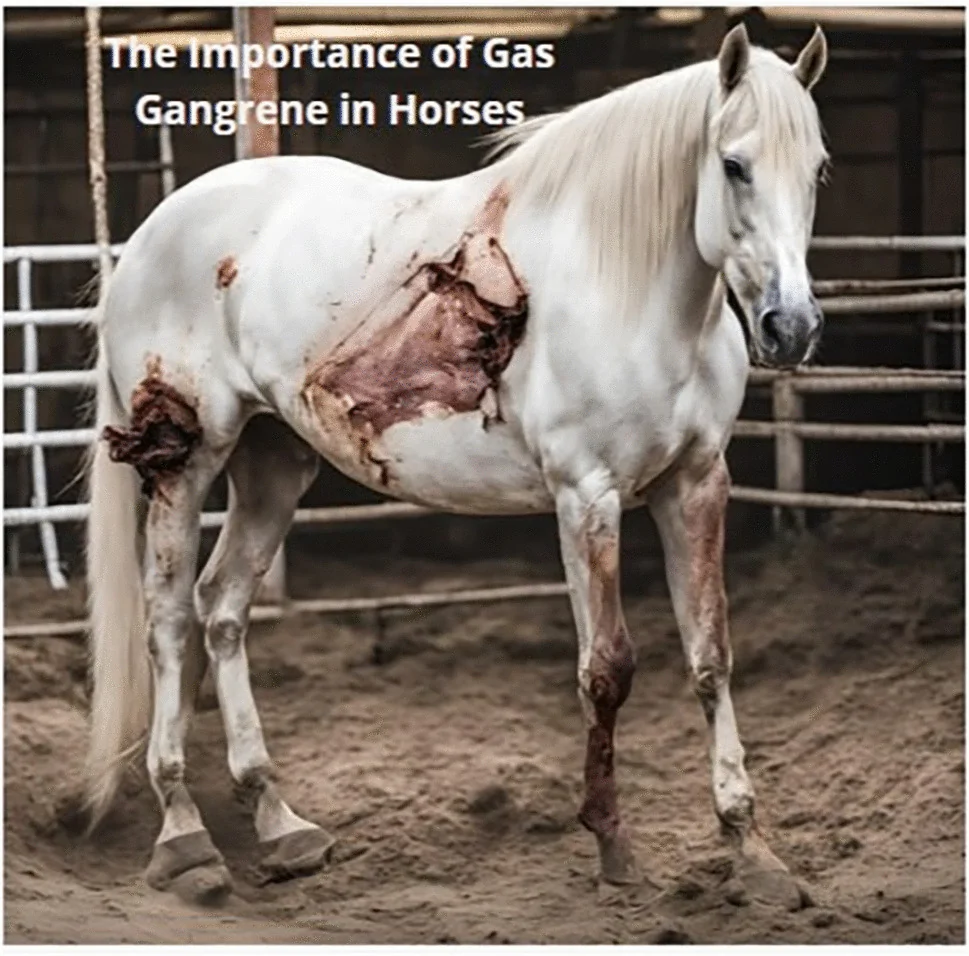

**Supplementary Information:**

The online version contains supplementary material available at https://doi.org/10.1007/s11259-026-11497-6.

## Introduction

Brazil hosts one of the largest equine populations worldwide, and horses remain central to multiple production and service chains, making severe infectious syndromes with high lethality particularly relevant to veterinary practice and animal health management (MAPA 2021). Among these syndromes, clostridial myonecrosis (“gas gangrene”) stands out as a peracute condition marked by extensive muscle necrosis, toxemia, and frequent death, demanding rapid clinical suspicion and prompt etiological confirmation. In horses, myonecrotic infections are classically linked to histotoxic clostridia that reach deep tissues through penetrating trauma or iatrogenic breaches in asepsis, including intramuscular injections and surgical procedures (Uzal et al. 2022; Junior et al. 2020). Although different agents have been implicated, fulminant courses and high case fatality are consistently reported, reinforcing the diagnostic urgency of this entity in equine medicine (Uzal et al. 2022).

Published evidence from tropical regions, particularly the Amazon biome, remains limited, despite ecological and management features that may facilitate exposure to clostridial spores and create opportunities for anaerobic proliferation in damaged tissues. The Amazon region is characterized by wide geographic dispersion of farms, heterogeneous veterinary coverage, and logistical constraints that may delay diagnosis and laboratory confirmation, thereby restricting case documentation in the peer-reviewed literature (Salvarani et al. 2025). In addition, preventive options for equine clostridial myonecrosis are not standardized; while vaccination strategies exist for other clostridial diseases, commercially available products specifically designed to protect horses against the main histotoxic species involved in gas gangrene are limited, emphasizing the role of basic biosecurity, injection hygiene, and wound management (Uzal et al. 2022; Junior et al. 2020).

Here, we report a clinicopathological and molecularly confirmed case series of 20 horses with gas gangrene from the eastern Brazilian Amazon. The objective is to document the clinical presentation, gross and histological lesions, and multiplex PCR findings, providing region-specific diagnostic evidence and practical insights for prevention focused on management-related risk points. Molecular confirmation is particularly relevant in this context because culture-based diagnosis may be compromised by post-mortem changes or prior antimicrobial exposure, and histopathology alone is not agent-specific (Uzal et al. 2022). This series also complements previous regional reports of acute equine myonecrosis linked to histotoxic clostridia in Brazil (Farias et al. 2014) and supports improved awareness of fatal clostridial myonecrosis in tropical equine systems.

Despite the recognized severity of clostridial myonecrosis in horses, systematic clinicopathological documentation from tropical production systems remains limited. Most available reports consist of isolated case descriptions or small outbreaks reported in temperate regions, and information regarding the occurrence, etiological agents, and pathological features of equine gas gangrene in the Amazon biome is scarce. Therefore, documenting well-characterized case series from this region is relevant to improve diagnostic awareness and to identify management practices that may predispose horses to this rapidly fatal infection.

## Case series description

### Case identification and inclusion criteria

A series of 20 horses with clostridial gas gangrene was identified between January 2018 and January 2026 in the eastern Amazon region of Brazil. Cases were retrieved from veterinary diagnostic laboratory records, clinical reports from field veterinarians, and necropsy submissions originating from several municipalities in the state of Pará. Only cases with compatible clinical history, characteristic gross lesions, histopathological findings consistent with clostridial myonecrosis, and positive multiplex polymerase chain reaction (PCR) detection of histotoxic clostridia were included in the final series. These 20 horses represented all suspected cases of clostridial myonecrosis identified during the study period that met the clinical and pathological criteria described above; all 20 cases were successfully tested by multiplex PCR and yielded positive results for *C. septicum*. Therefore, no suspected cases were excluded from the series because of unsuccessful or unavailable PCR results.

To ensure diagnostic consistency, cases were included only when clinical, pathological, and molecular criteria were concurrently fulfilled. Clinically, horses presented with sudden onset of severe localized swelling, marked pain on palpation, and palpable subcutaneous crepitus, frequently accompanied by systemic manifestations compatible with toxemia such as fever, lethargy, or anorexia. Gross pathological findings obtained during necropsy or surgical exploration consistently revealed extensive hemorrhagic myonecrosis with intramuscular gas formation, serosanguineous exudation, and a characteristic foul odor. Molecular confirmation of *Clostridium septicum* was performed using a multiplex PCR assay targeting major histotoxic clostridia, including *Clostridium chauvoei, C. septicum, C. novyi* type A*, C. perfringens* typ*e* A, and *Paraclostridium sordellii*, following the protocol described by Ribeiro et al. (2012). A DNA mixture containing DNA from the reference strains C*. chauvoei* ATCC 10092, *C. septicum* ATCC 12464, *P. sordellii* ATCC 9714, *C. novyi* type A ATCC 19402, and *C. perfringens* type A ATCC 3624 was used as the positive control. A no-template control containing all PCR reagents, with nuclease-free water replacing template DNA, was included as the negative control.

### Clinical presentation and epidemiological context

The affected horses originated from five municipalities in the state of Pará, including Castanhal, Novo Progresso, Santo Antônio do Tauá, São Félix do Xingú, and Soure (Marajó Island). Animals ranged from 4 months to 12 years of age, with a predominance of adult individuals. Most cases involved male horses.

Clinical evolution was characterized by rapidly progressive swelling of affected limbs or muscle groups, severe lameness, intense pain, and palpable subcutaneous emphysema. Systemic deterioration occurred rapidly, with tachycardia, tachypnea, and signs compatible with systemic toxemia frequently observed during clinical examination, performed according to standard equine semiological guidelines (Feitosa 2020). The main clinical and epidemiological findings observed in the 20 affected horses are summarized in Table 1.

**Table 1:** Main clinical and epidemiological findings observed in horses with clostridial gas gangrene in the Brazilian Amazon

Clinical/epidemiological variable	Number of cases (n = 20)	Frequency (%)
Severe lameness	15/20	75%
Intense localized pain	20/20	100%
Rapidly progressive swelling	15/20	75%
Subcutaneous crepitus/emphysema	20/20	100%
Systemic signs compatible with toxemia	18/20	90%
Previous intramuscular injection	15/20	75%
Penetrating trauma	3/20	15%
Minor surgical procedure	2/20	10%
Death/euthanasia	20/20	100%

In all animals, a recent breach in tissue integrity was reported prior to the onset of clinical signs. These events included intramuscular injections, minor surgical procedures, or penetrating traumatic injuries, including skin lesions associated with environmental hazards such as barbed wire or wooden structures. Because of the retrospective nature of the study, the specific medications administered intramuscularly could not be reliably retrieved from the available clinical records. In several farms, management practices potentially favoring contamination were documented, including reuse of needles and inadequate sterilization of instruments. None of the affected horses had a documented history of vaccination against clostridial diseases.

**Fig. 1:**
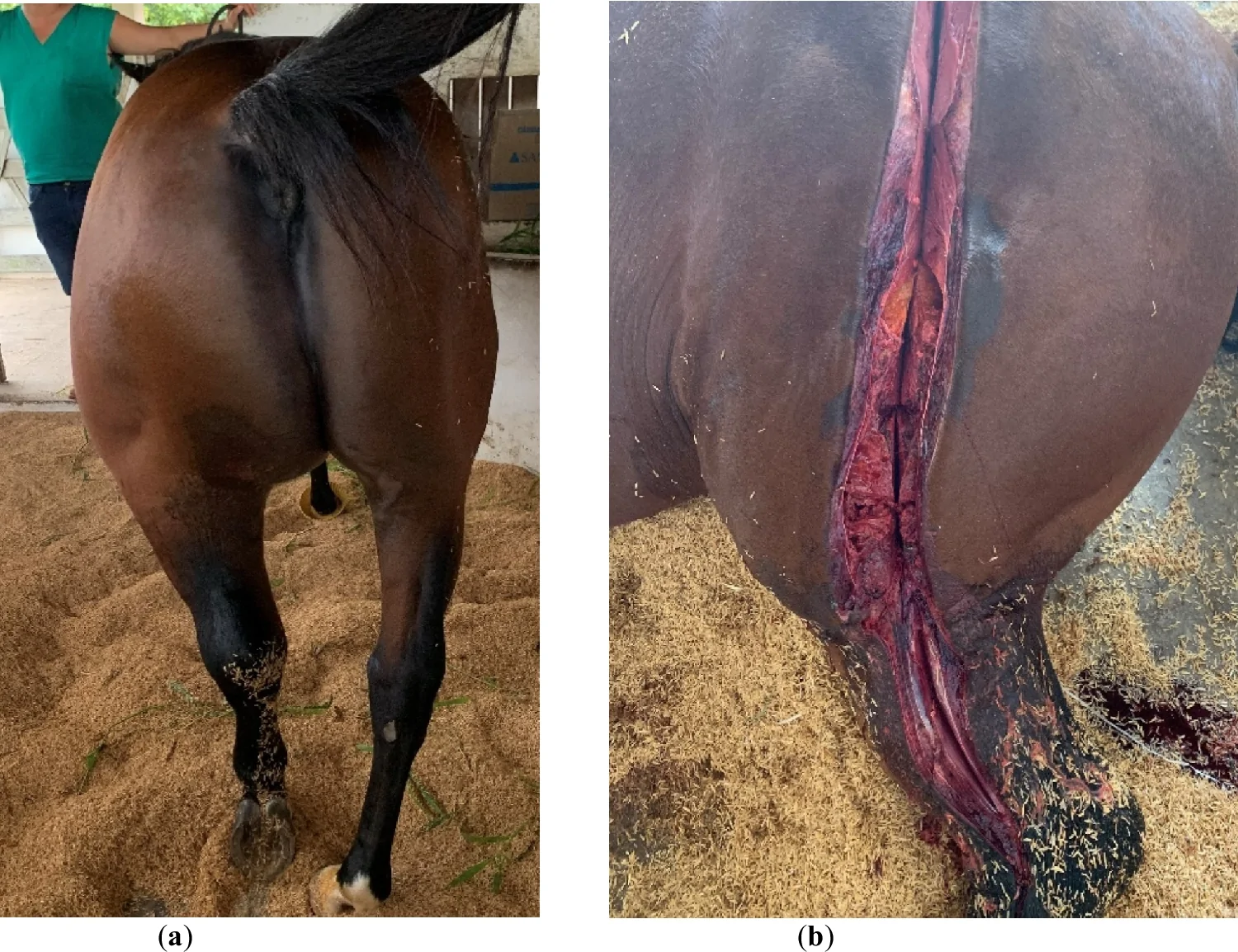
Equine with gas gangrene: (**a**) increased volume of the left hind limb; (**b**) hemorrhage and subcutaneous edema, with extravasation of serosanguineous fluid, liquefaction, and tissue necrosis (post-mortem)

**Fig. 2:**
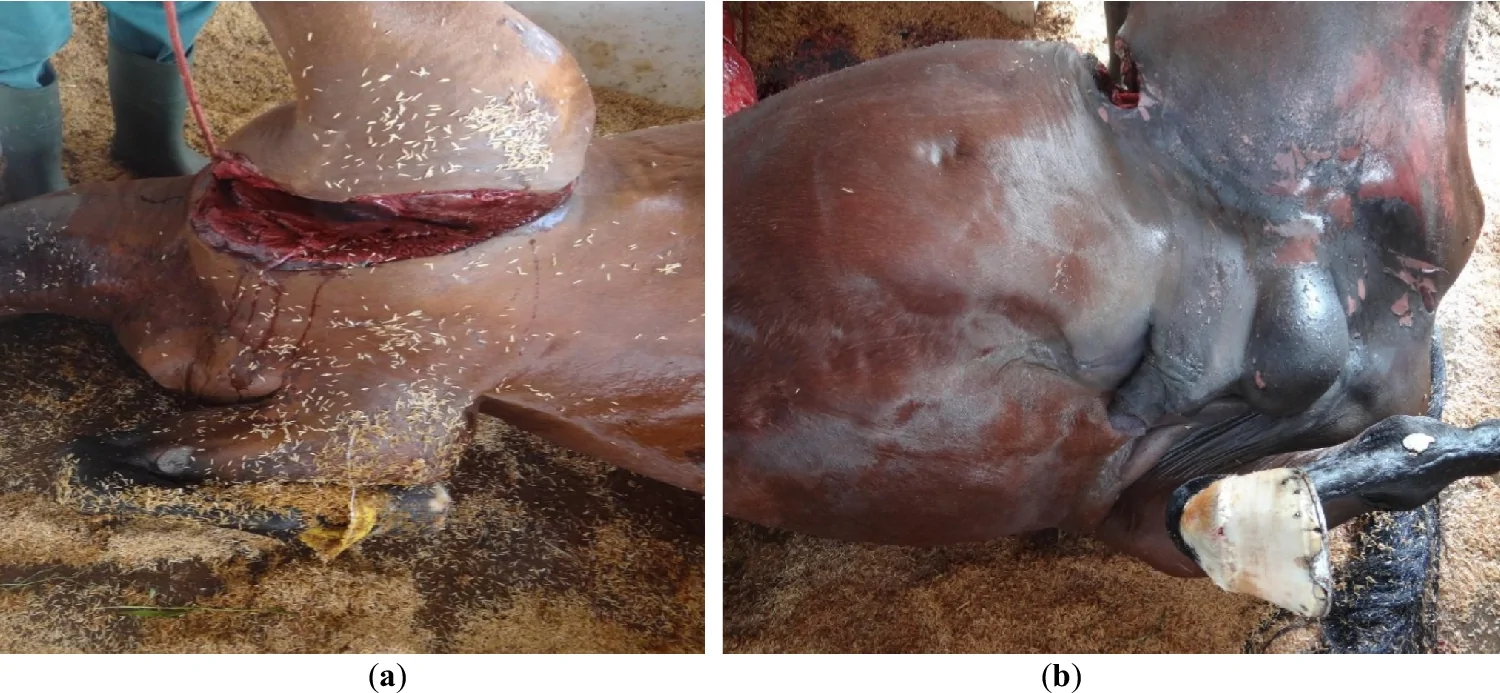
Equine with gas gangrene (postmortem): (**a**) hemorrhage and subcutaneous edema, with extravasation of serosanguineous fluid, liquefaction, and tissue necrosis in the pectoral region; (**b**) ventral subcutaneous edema

Despite administration of antimicrobial therapy and anti-inflammatory treatment, clinical deterioration progressed rapidly in all animals. The case fatality rate reached 100%, with horses dying or being euthanized due to poor prognosis within a short period after the onset of clinical signs. Representative clinical and gross pathological findings are illustrated in Fig. 1, which demonstrates marked enlargement of the affected limb with extensive subcutaneous edema and hemorrhage, and Fig. 2, showing diffuse muscular necrosis with serosanguineous fluid accumulation and tissue liquefaction.

### Pathological examination

Necropsy examinations consistently revealed lesions typical of clostridial myonecrosis. Affected musculature displayed extensive hemorrhagic necrosis with interstitial edema and accumulation of serosanguineous fluid. Gas bubbles were frequently present within muscle fascicles and subcutaneous tissues, producing a characteristic crepitant texture.

Histopathological evaluation was performed on representative tissue samples collected during necropsy or surgical intervention. Samples were fixed in buffered formalin, routinely processed, embedded in paraffin, and stained with hematoxylin and eosin following standard histological procedures (Amorim et al. 2011).

Microscopically, lesions were characterized by severe coagulative to floccular necrosis of skeletal muscle fibers, associated with extensive interstitial edema and hemorrhage. Muscle fibers frequently exhibited fragmentation and loss of cross-striations, often replaced by eosinophilic amorphous debris. Inflammatory infiltrates composed predominantly of neutrophils were present in necrotic areas. Vascular thrombosis was observed in several cases. Numerous intralesional bacillary structures morphologically compatible with clostridia were observed within areas of myonecrosis in all cases. The principal microscopic findings observed in the case series are summarized in Table 2.

**Table 2:** Main histopathological findings observed in horses with clostridial gas gangrene in the Brazilian Amazon

Histopathological finding	Number of cases (n = 20)	Frequency (%)
Severe coagulative and/or floccular myonecrosis	20	100
Intramuscular gas vacuoles	20	100
Diffuse interstitial edema	20	100
Intramuscular hemorrhage	20	100
Neutrophil-predominant inflammatory infiltrate	20	100
Intralesional bacillary structures	20	100
Vascular thrombosis	16	80
Muscle fiber fragmentation and loss of cross-striations	20	100
Centrilobular hepatocellular vacuolization	6	30

**Fig. 3:**
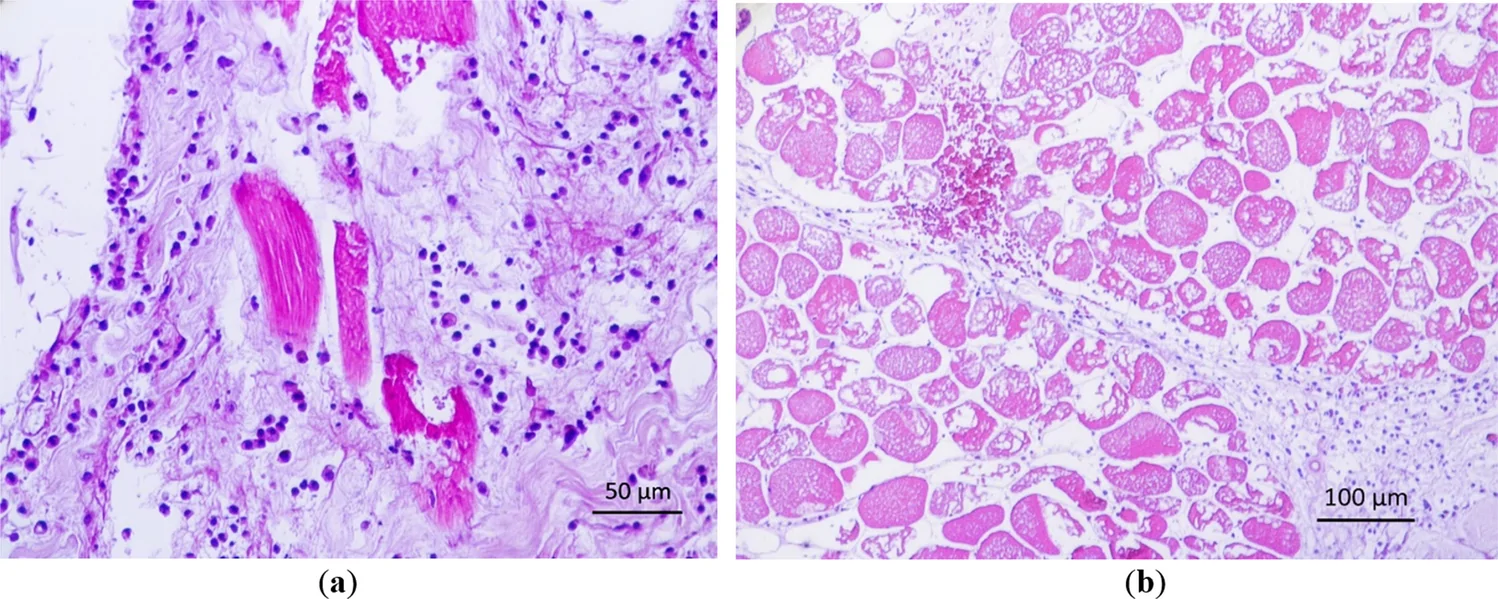
Histopathological findings in equine gas gangrene. **a** Severe degeneration and necrosis of skeletal muscle fibers associated with inflammatory cell infiltration and interstitial edema. H&E. Bar = 50 µm. **b** Multifocal to coalescing myofiber degeneration and necrosis with marked interstitial edema and inflammatory infiltration. H&E. Bar = 100 µm

Representative histopathological lesions are shown in Fig. 3, illustrating extensive degeneration and necrosis of muscle fibers associated with inflammatory infiltrates, edema, and hemorrhage.

### Molecular findings

Multiplex PCR successfully amplified *C. septicum*-specific DNA from affected skeletal muscle samples from all 20 horses (20/20; 100%). No amplification was detected for *C. chauvoei*, *C. novyi* type A, *C. perfringens* type A, or *P. sordellii* in any of the samples analyzed. The positive control yielded the expected amplification products, whereas no amplification was observed in the no-template negative control.

## Discussion and conclusions

Necrotizing clostridial infections are among the most aggressive bacterial diseases affecting equine skeletal muscle (Vengust et al. 2003). The present case series provides clinicopathological and molecular documentation of 20 fatal cases of equine gas gangrene associated with *C. septicum* diagnosed in the eastern Brazilian Amazon. The most notable observation was the consistent molecular detection of *C. septicum* in all examined animals, combined with a remarkably uniform clinical and pathological presentation. Although several histotoxic clostridia have been implicated in equine gas gangrene, including *C. perfringens, C. novyi, C. chauvoei,* and *P. sordellii*, infections attributed specifically to *C. septicum* remain comparatively less frequently documented in horses (Uzal et al. 2022; Junior et al. 2020; Peek et al. 2003). Consequently, the present series contributes valuable documentation of the involvement of this species in fatal clostridial myonecrosis affecting horses raised under tropical production conditions. The consistent detection of *C. septicum* across all 20 cases provides evidence of its involvement in equine clostridial myonecrosis in the studied region. However, because this case series was not designed to estimate pathogen prevalence or compare the relative frequency of histotoxic clostridial species, no inference regarding the predominance of *C. septicum* in the Amazon biome can be made from these data.

The clinical evolution observed in the affected animals closely corresponds to classical descriptions of clostridial gas gangrene. The abrupt onset of severe lameness, rapidly progressive swelling, and palpable subcutaneous crepitation reflects the extensive tissue destruction and gas production generated during anaerobic bacterial proliferation in damaged muscle tissue (Uzal et al. 2022). The uniformly fatal outcome documented in this series further highlights the fulminant nature of this condition. Previous clinical studies have reported mortality rates ranging from approximately 50% to nearly 100% in equine clostridial myonecrosis, depending on the time of diagnosis and the possibility of aggressive surgical intervention (Peek et al. 2003; Junior et al. 2020). In the present cases, despite the administration of antimicrobial and anti-inflammatory therapy, disease progression was extremely rapid, emphasizing the narrow therapeutic window that characterizes this infection.

The gross and microscopic lesions observed in the affected horses were consistent with the well-recognized pathological features of clostridial myonecrosis. Extensive hemorrhagic necrosis of skeletal muscle, diffuse edema, intramuscular gas accumulation, and intralesional bacillary structures are characteristic pathological features of clostridial myonecrosis (Amorim et al. 2011; Uzal et al. 2022). Histopathological uniformity across all animals examined strengthens the diagnostic consistency of the case definition and provides biological support for the molecular results obtained in the present study. The frequent observation of vascular thrombosis and widespread muscle fiber degeneration further reflects the combined effects of bacterial toxins, tissue ischemia, and host inflammatory responses, which together contribute to the rapid clinical deterioration typical of clostridial infections.

An important epidemiological observation in this series was the association of 15 of 20 cases (75%) with previous intramuscular injections, while the remaining cases were associated with penetrating trauma or minor surgical procedures. These conditions are widely recognized as facilitating the introduction of clostridial spores into muscle tissue. Several classical reports have documented similar iatrogenic or traumatic entry points in equine gas gangrene outbreaks, particularly following intramuscular injections or minor surgical procedures (Breauhaus et al. 1983; Peek et al. 2003). In the present cases, additional management practices potentially contributing to infection risk were identified, including reuse of needles and inadequate sterilization of instruments. Although causal relationships cannot be definitively established in an observational case series, these findings highlight important opportunities for preventive intervention through improved aseptic practices and veterinary supervision.

The exclusive molecular detection of *C. septicum* in all animals is noteworthy. This species has been recognized as an important cause of malignant edema and spontaneous myonecrosis in several animal species (Raymundo et al. 2010; Junior et al. 2020; Uzal et al. 2022). In horses, however, reports of *C. septicum*–associated gas gangrene remain limited compared with those involving *C. perfringens* type *A* or other histotoxic clostridia (Kennedy et al. 2009; Peek et al. 2003; Sacco et al. 2020). While the consistent identification of *C. septicum* in this series suggests that this pathogen may play a relevant role in equine clostridial infections within the studied region, caution is required in interpreting this observation. The retrospective nature of the study, the limited number of cases, and the targeted PCR approach preclude definitive conclusions regarding pathogen predominance in the Amazon biome. Nevertheless, the occurrence of molecularly confirmed cases across several municipalities demonstrates that *C. septicum*-associated equine myonecrosis was not restricted to a single locality within the studied region.

Environmental factors may influence the epidemiology of clostridial infections, and the persistence of clostridial spores in soil represents a potential source of exposure (Junior et al. 2020). However, the present study did not investigate environmental reservoirs or climatic factors, and therefore no association between the environmental conditions of the Amazon biome and the occurrence of the disease can be inferred from these cases. Future studies incorporating environmental sampling and molecular epidemiological approaches would be valuable to investigate potential reservoirs and transmission pathways of histotoxic clostridia in tropical equine systems.

The absence of vaccination against clostridial diseases in all affected animals deserves consideration, although its interpretation must remain cautious. Vaccination strategies targeting several clostridial toxins have been developed for livestock species and experimental vaccine approaches have been explored in horses (Zaragoza et al. 2019; Freitas et al. 2021). However, protective protocols specifically designed to prevent equine gas gangrene remain limited, and the effectiveness of available vaccines against multiple histotoxic clostridia in horses is still incompletely defined (Uzal et al. 2022). Consequently, improvements in management practices, particularly strict aseptic techniques during injections and prompt treatment of traumatic wounds, remain the most practical preventive strategies currently available.

From a diagnostic perspective, the integration of clinical evaluation, pathological examination, and molecular detection proved particularly valuable in confirming the etiological diagnosis. Culture-based identification of clostridia may be compromised in field conditions due to post-mortem autolysis or prior antimicrobial therapy, which frequently occur in severe cases of myonecrosis (Ribeiro et al. 2012; Uzal et al. 2022). In this context, multiplex PCR represents a useful complementary diagnostic tool capable of detecting specific clostridial species directly in affected tissues.

Some limitations inherent to this study should be acknowledged. The retrospective design restricts the availability of complete exposure data, including reliable information on the specific medications administered in cases associated with intramuscular injections, and the case-series design precludes robust epidemiological inference. In addition, toxin characterization and bacterial isolation were not consistently achieved in all animals. Nevertheless, the strong concordance between clinical signs, pathological findings, and molecular detection provides reliable evidence supporting the diagnosis of clostridial gas gangrene associated with *C. septicum*. From a practical perspective, these observations emphasize that even minor breaches in tissue integrity may create conditions for clostridial proliferation in equine muscle, particularly in environments where spores are likely to persist in soil and organic matter. However, to the best of our knowledge, this is one of the largest documented case series of equine gas gangrene associated with *Clostridium septicum* reported in South America.

In summary, this case series documents 20 fatal cases of equine gas gangrene associated with *C. septicum* in the Brazilian Amazon and provides clinicopathological and molecular evidence supporting the involvement of this pathogen in equine clostridial myonecrosis under tropical conditions. The findings reinforce the fulminant nature of the disease, highlight the value of molecular diagnostics for etiological confirmation, and underscore the importance of strict aseptic practices and early clinical recognition in equine management systems.

## Supplementary Information

Below is the link to the electronic supplementary material.

Supplementary file1 (PDF 195 KB)

Supplementary file2 (PDF 745 KB)

## Data Availability

Data are contained within the article.
