# Supplementary material for: Fatal equine gas gangrene associated with Clostridium septicum in the Brazilian Amazon: clinicopathological and molecular characterization of a 20-case series
Source: Vet Res Commun. 2026 Sep 4;50(6):551. doi: 10.1007/s11259-026-11497-6 (PMC13545102; doi:10.1007/s11259-026-11497-6)
Supplement: Supplementary file 1 [file 11259_2026_11497_MOESM1_ESM.pdf]

UNIVERSIDADE FEDERAL DO PARÁ
INSTITUTO DE MEDICINA VETERINÁRIA
HOSPITAL VETERINÁRIO

CONSENT TERM AND ENLIGHTENMENT TO PERMISSION FOR DATA COLLECTION AND APPLICATION

College professor/Researcher responsible: Felipe Masiero Salvarani
Aim of study: Study of infectious, deficiency, metabolic, genetic and immunological diseases in animals in the Amazon Biome
Potential risks to animals: not applicable
Timeline: 2023/2025

ENLIGHTENMENT TO THE OWNER ABOUT THE ANIMAL'S PARTICIPATION IN THIS STUDY/PROJECT

Your authorization to include your animal(s) in this study is voluntary. Your animal(s) may be withdrawn from the study at any time without causing any harm to you and/or them. The confidentiality of your personal data will be preserved. The Veterinarian responsible for your animal(s) will be **Loise Araújo de Sousa**, registered with the **CRMV number 8201**. In addition to him, the team of **Professor/Researcher Dr. Felipe Masiero Salvarani**, who will also be responsible fot the well-being of your animal(s) throughout the study auntil its end. When necessary, during or after the study period, you can contact the principal researcher or his team through the contacts:
Emergency Phone: +55 (91) 993173503
Team: Prof. Dr. Felipe Masiero Salvarani

CONSENT TERM

I, _______________, ownership/legal guardianship of the animal(s) _________________, declare that I have been duly informed about all the procedures of this study, its risks and benefits to the animal(s) which(es) I am responsible. I was also informed that I can withdraw my animal(s) from the study at any time. By signing this consente form, I declare that I authorize the participation of my animal(s), identified below, in this studY and the use of this study results for future publications.

Ownership name/Legal guardianship of animal(s):
Cadastro de pessoa física (CPF)

Animal(s) identification (repeat the table as many times as the number of animals of the same ownership/legal guardianship).

**Name:**		**Identification Number:**
**Species:**	**Breed:**	**Gender:**

This document will be signed in two copies, one copy will be with the legal guardianship and the Other with the responsible professor/researcher.

(City), Date(day/month/year)

Responsible Professor/Researcher signing

Ownership/Legal Guardianship signing

.
