# Supplementary material for: Fatal equine gas gangrene associated with Clostridium septicum in the Brazilian Amazon: clinicopathological and molecular characterization of a 20-case series
Source: Vet Res Commun. 2026 Sep 4;50(6):551. doi: 10.1007/s11259-026-11497-6 (PMC13545102; doi:10.1007/s11259-026-11497-6)
Supplement: Supplementary file 2 [file 11259_2026_11497_MOESM2_ESM.pdf]

Editing Certificate

This document certifies that the manuscript

Fatal equine gas gangrene associated with Clostridium septicum in the Brazilian Amazon: clinicopathological and molecular characterization of a 20-case series

prepared by the authors

Loise Araújo de Sousa, Laura Rosa Corrêa, Hanna Gabriela da Silva Oliveira and Felipe Masiero Salvarani

was edited for proper English language, grammar, punctuation, spelling, and overall style by one or more of the highly qualified native English speaking editors at AJE.

This certificate was issued on March 04, 2026 and may be verified on the AJE website using the verification code 89PL-4GY7-9E4R-L12B-Y4TT.

Neither the research content nor the authors' intentions were altered in any way during the editing process. Documents receiving this certification should be English-ready for publication; however, the author has the ability to accept or reject suggestions and changes. To verify the final AJE edited version, please visit our verification page at aje.com/certificate If you have any questions or concerns about this edited document, please contact AJE at support@aje.com.

AJE provides a range of editing, translation, and manuscript services for researchers and publishers around the world.

For more information about our company, services, and partner discounts, please visit aje.com
